# H3K18 lactylation in pancreatic cancer: promising yet unproven ‒ a call for validation

**DOI:** 10.1016/j.clinsp.2025.100715

**Published:** 2025-06-24

**Authors:** Sohaib Aftab Ahmad Chaudhry, Rida Shakeel, Javed Iqbal

**Affiliations:** a Department of Surgery, ABWA Medical College, Faisalabad, Pakistan; b Department of Surgery, Dow Medical College, Karachi, Pakistan; c Department of Nursing, Hamad Medical Corporation, Doha, Qatar

We read with interest the recent article by Hou et al. published in *Clinics* which proposed lactylated histone H3K18 (H3K18la) as a biomarker for diagnosis and severity prediction of pancreatic cancer.^1^ With pancreatic cancer’s 5-year survival rate languishing below 12 %, early detection remains a critical unmet need.^2^ The authors’ emphasis on H3K18 lactylation, a metabolic-epigenetic modification linked to the Warburg effect, is a novel approach that can potentially improve outcomes.^3^ However, for H3K18la to be adapted into clinical practice, it would have to go through extensive testing since the current study’s shortcomings leave much to be answered.

The study’s underlying idea is compelling: H3K18la concentration is elevated in Pancreatic Ductal Adenocarcinoma (PDAC) tissues and may be linked to tumor aggressiveness. This is line with the evidence that lactate-induced epigenetic modifications influence cancer progression.^3^ However, the methodology raises concerns. The sample size seems inadequate, and the absence of a multicenter cohort makes extrapolating findings over the general population difficult.^4^ The genetic heterogeneity of pancreatic cancer highlights the need for a comprehensive validation that has been unaddressed in the article.^4^ Moreover, the experiment doesn’t deal with specificity ‒ H3K18la increase may overlap with inflammatory conditions like chronic pancreatitis, a frequent confounder in PDAC diagnosis. Without sensitivity and specificity data, ideally through a Receiver Operating Characteristic (ROC) analysis, the diagnostic role of H3K18la remains theoretical.^5^

The predictive claim ‒ linking H3K18la to the severity of the disease ‒ has not been established either. The authors point out its connection with the advanced stages of the tumor, but the statistical method used is unclear. Have they considered the impact of PDAC risk factors like diabetes and smoking in their statistics? Moreover, the dependence on tissue samples limits practical application. Blood-based biomarkers are better for early screening as evidenced by trials such as the NAPOLI-3 trial.^6^ These gaps illustrate the unreliability of H3K18la, despite its theoretical promise.

To address these queries, we request a call for validation. Future investigations ought to prioritize larger, multicenter cohorts to ensure the reproducibility of results, as well as emphasis on specificity testing to differentiate PDAC from benign conditions. The adoption of liquid biopsies could improve clinical feasibility, in line with the precision oncology trends as observed in the POLO trial.^7^ The promise of H3K18la can only be realized through validation in a comprehensive manner, potentially leading to a paradigm shift in pancreatic cancer management.

## Funding

This research did not receive any specific grant from funding agencies in the public, commercial, or not-for-profit sectors.

## Declaration of competing interest

The authors declare no conflicts of interest.
